# Understanding how enzymes work: the journey to ensemble–function studies

**DOI:** 10.1111/febs.70456

**Published:** 2026-02-18

**Authors:** Daniel Herschlag, Siyuan Du

**Affiliations:** ^1^ Department of Biochemistry Stanford University California USA; ^2^ Stanford ChEM‐H, Stanford University CA USA; ^3^ Department of Chemical Engineering Stanford University CA USA; ^4^ Department of Chemistry Stanford University CA USA

**Keywords:** conformational ensemble, energy landscape, enzyme catalysis, enzyme positioning, ground state destabilization, statistical mechanics, structure–function

## Abstract

In this perspective, we describe how we arrived at a framework of ensemble–function analyses to quantitatively dissect enzyme catalysis and biological function more broadly. Serine proteases are described in biochemistry textbooks to illustrate enzyme mechanisms, yet those descriptions do not explain *how* these enzymes achieve their ~ 10^12^‐fold rate enhancements. Moving away from the classic descriptions of ‘catalytic triad’ and ‘oxyanion hole’, we returned to the basic physical and chemical interactions in serine protease active sites and identified molecular features that enable a highly efficient reaction path on the enzymes, compared to the uncatalyzed reaction. We then leveraged principles from statistical mechanics to quantify the contributions from each catalytic feature. Combining the contributions from each feature in a ‘catalytic ledger’ provided a quantitative accounting of serine protease catalysis. These analyses revealed previously unrecognized catalytic interactions that are destabilizing in the reaction's ground state—unfavorable bond rotamers, shorter‐than‐ideal distances, and suboptimal hydrogen bonds—each of which is relieved in the transition state, thereby lowering the barrier to reaction. Analogous catalytic features are found in over 30 different protease and nonprotease enzymes spread across 12 structural folds, suggesting that nature has taken advantage of these strategies multiple times in different contexts. In the future, ensemble–function analyses can be used to derive quantitative mechanistic models for other enzymes, to dissect allostery, and to ascertain how molecular machines operate. Ensemble–function also provides a powerful educational approach by linking the complex behavior of biomolecules to the simple chemical and physical principles that are taught in undergraduate classes.

## Graphical abstract

For decades, structure–function has dominated biochemistry. Structures are highly valuable, yet more is needed to achieve a quantitative understanding of biomolecular function, because function emerges from an ensemble of states, rather than a static structure. We describe an ensemble–function framework applied to quantitatively dissect serine protease catalysis. This framework provides quantitative mechanisms of biological functions grounded in basic chemical and physical principles, an approach that can transform biochemical research and education.

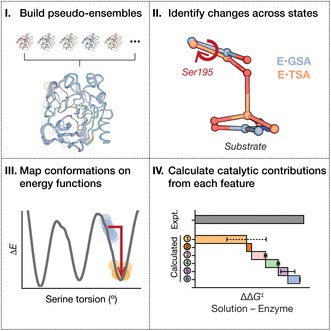

**Abbreviations**
GS: ground state
GSA: ground state analog
TS: transition state
TSA: transition state analog

If one were to ask students or researchers in molecular biology or biochemistry which enzyme we really understand, many would reply ‘serine proteases’, as they are—literally—the textbook example for enzyme catalysis. Biochemistry textbooks clearly elucidate the steps in the reaction cycle and the residues involved in each step (Fig. 1A), and mutating these residues is highly deleterious to catalysis [1, 2]. The same residues and chemical groups have emerged in serine proteases from distinct structural folds, a remarkable example of convergent evolution [3]. Yet, while the chemical mechanism is well defined, textbook descriptions do not illuminate *how* serine proteases achieve their enormous rate enhancements. Figure 1B compares the properties of each catalytic group in serine proteases highlighted in textbooks with the corresponding interactions in the aqueous solution: The serine nucleophile is a hydroxyl group and thus not more reactive than water, the oxyanion hole amide groups are not intrinsically stronger hydrogen bond donors than water, and the catalytic triad, while providing a histidine general base, falls far short of accounting for the full ~ 10^12^‐fold rate advantage obtained by serine proteases [4]. What, then, are the features or properties that underlie the remaining serine protease catalysis?

**Fig. 1:**
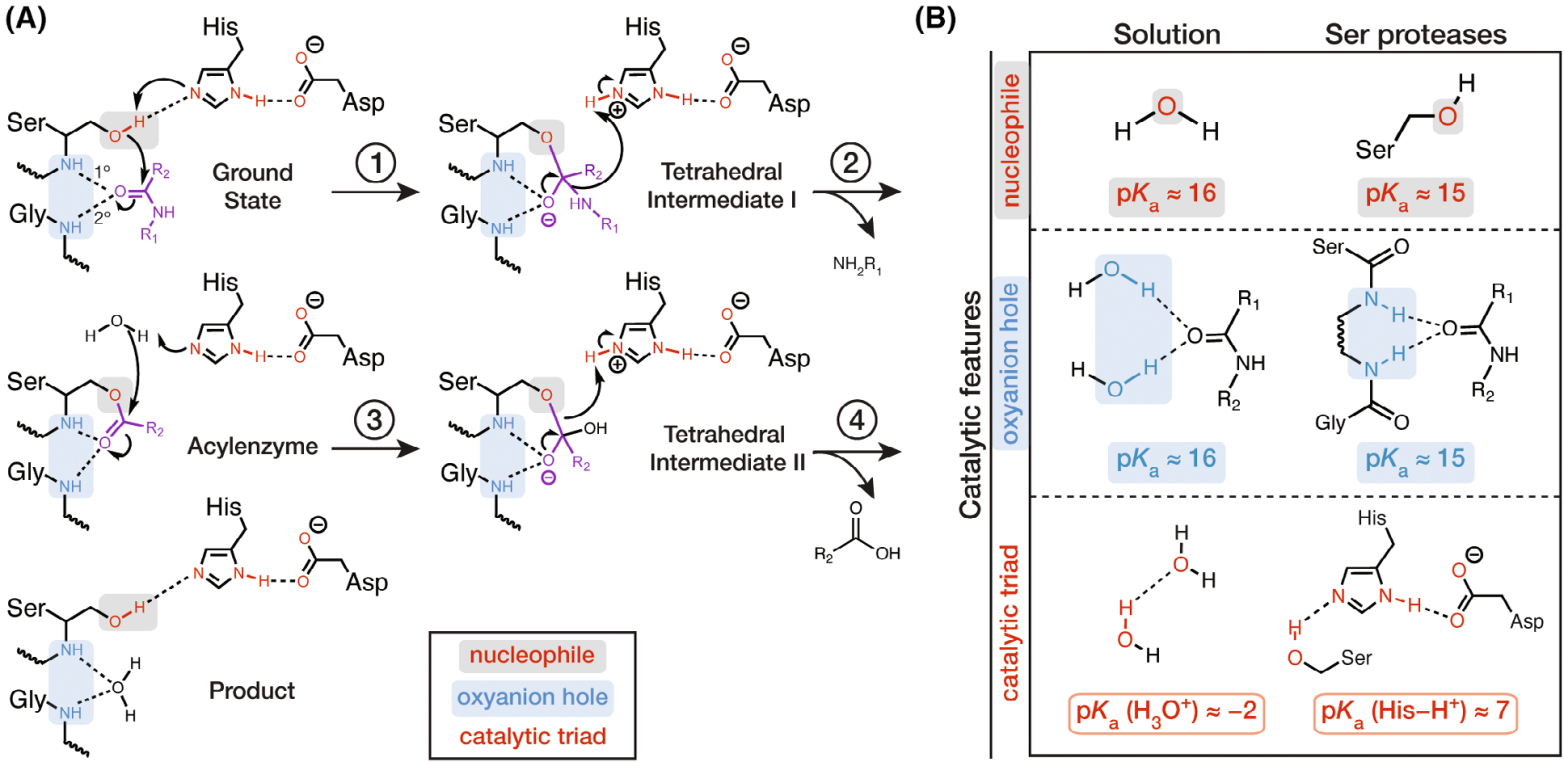
The serine protease reaction and active site groups. Adapted from ref. [4] with permission. (A) The chemical mechanism of the serine protease reaction. The hydrogen bonding atoms of the catalytic triad are colored red; the nucleophile is shaded in gray; the oxyanion hole hydrogen bond donor atoms are colored and shaded light blue; and the substrate amide is purple. (B) Chemical features of the enzyme catalytic groups compared with analogous interactions in the solution reaction. p*K*_a_ values are used as proxies for electron density [55, 56, 57], using the following values from ref [58]: intrinsic p*K*_a_ values of water and hydronium ion; acetamide for the oxyanion hole donors; methanol for the serine side chain; and imidazole for the histidine side chain.

Perhaps the inability to identify and quantify catalytic strategies in serine proteases arises from something fundamental to our approach. Galileo recognized centuries ago that transitioning from qualitative descriptions to quantitative models demands a different ‘language’, a language that he argued was needed for meaningful scientific understanding. In 1623, when considering the laws of the universe, he wrote:

‘[The universe] cannot be understood unless one first learns to comprehend the language and read the letters in which it is composed. It is written in the language of mathematics…without which it is humanly impossible to understand a single word of it; without these, one wanders about in a dark labyrinth’. [5]

The book of biology is also not written in words. Biology is constructed from molecules, and the natural language to describe molecules is chemistry and physics. Molecules are held together by the atomic‐level interactions—covalent bonds, hydrogen bonds, van der Waals and electrostatic interactions, etc.—which form the basic vocabulary of this language. The function of biomolecules, such as ligand binding and enzyme catalysis, is defined and quantified by the *probabilities* of forming, breaking, and altering these interactions, which are governed by the laws of statistical mechanics (Fig. 2). The vocabulary of atomic‐level interactions and the ‘grammar’ of statistical mechanics allowed us to discover catalytic features and quantify their contributions, arriving at a model that accounts for serine protease catalysis, as we describe below.

**Fig. 2:**
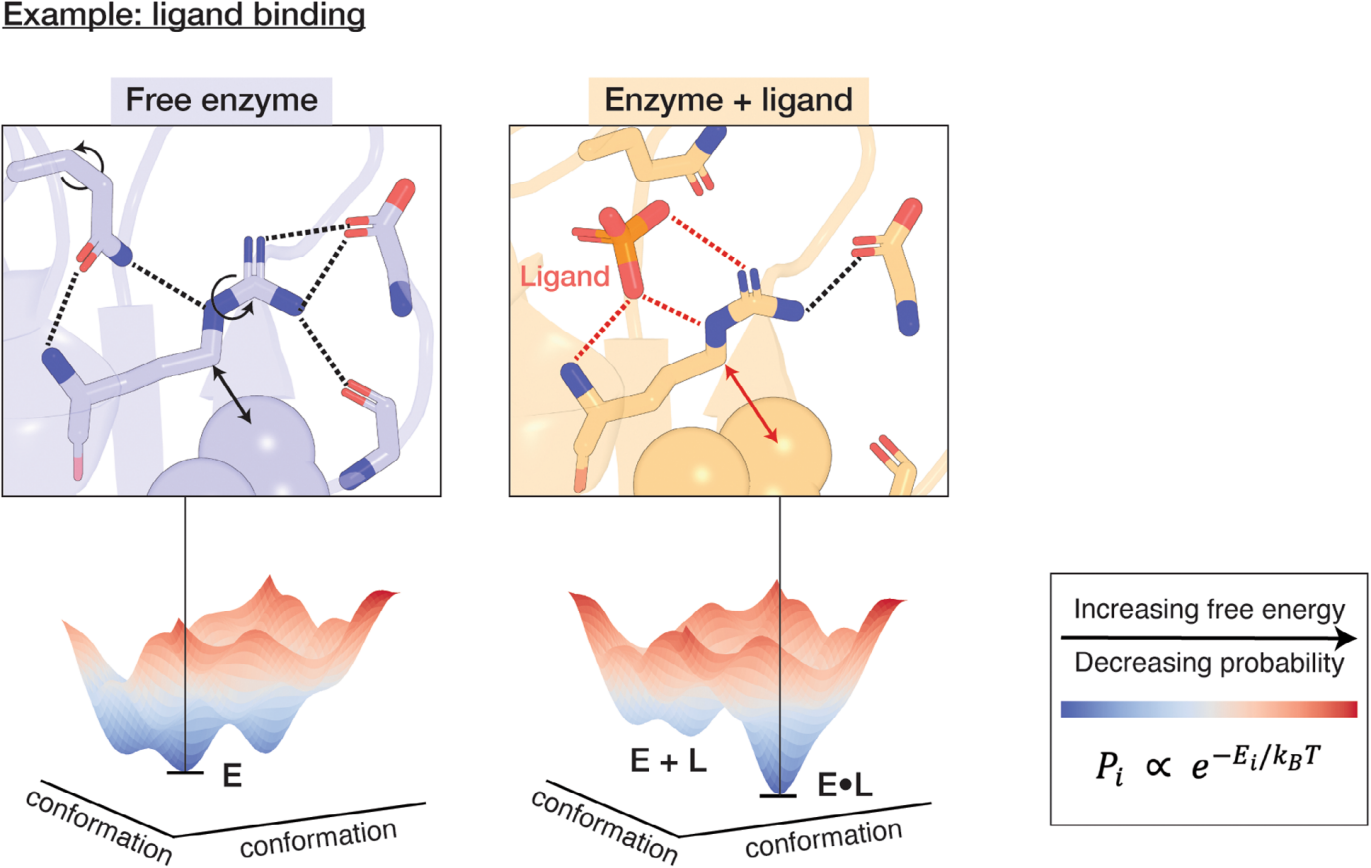
Equilibrium processes emerge from molecular interactions and their underlying energetics. A simple example for the binding of a ligand (L) to an enzyme (E) to form the E•L complex is shown. The free enzyme occupies an ensemble of conformational states, shown as a conformational landscape. The probability of occupying each state (*P*_i_) is governed by its energy (*E*_*i*_), as determined by the Boltzmann relationship. In a system with the enzyme and a ligand, the conformational landscape is modified, favoring the E•L state due to favorable binding interactions. The example shown is acylphosphatase, and for simplicity, only one favored free (PDB: 2FHM) [59] and bound state (PDB: 3BR8) [60] is shown in molecular representation. In this case, the predominant free and bound states shown have different bond torsions, hydrogen bonds, and van der Waals interactions. The binding affinity is defined as the relative free energy of the unbound and bound states, summing over all microstates that are unbound (E•L) and bound (E + L) in the ensemble.

## From structure–function to ensemble–function

Structure–function has been the dominant paradigm in biochemistry for decades. Before describing how we dissected enzyme catalysis by going beyond structure–function, we emphasize what is already widely appreciated—that structure has extraordinary value. As one of thousands of examples, consider the so‐called DNA clamp proteins that adopt ring structures with positively charged interiors and openings just large enough to accommodate double‐stranded DNA; their shape immediately tells us how they work as a DNA polymerase processivity factor [6]. Nevertheless, structure does not tell us the stability of the ring structure, how fast it opens and closes, or what controls its opening and closing to allow processive DNA synthesis to be initiated and terminated—parameters and properties of central importance for understanding its function. Single, static structures are not sufficient to obtain these functional parameters, because these parameters are defined by multiple protein states (folded *vs*. unfolded, open *vs*. closed, etc.) and their relative probabilities. The probability of each state is needed and is determined by their energies as defined in statistical mechanics (Fig. 2).

As for all other biomolecular processes, enzyme rates are quantified by the relative probability of states—the reaction ground state (GS) and transition state (TS). The amount of catalysis provided by enzymes is, therefore, defined by the *increased* probability of a reaction on the enzyme relative to that in solution. This increased probability corresponds to a reduced free energy barrier to reaction, which enzymes achieve by preferentially stabilizing the TS over the GS (see also fig. 1 of ref. [7]). This preferential stabilization is a definition of catalysis, not an explanation or mechanism. Where, then, do we start to figure out what enzyme features are responsible for the increased probabilities or preferential stabilization, and by how much?

Over the past century, there have been numerous conceptual and experimental advances in understanding enzyme catalysis. Polyani [8, 9] and Pauling [10] proposed that enzymes act by making favorable interactions that closely match the shape of the transition state rather than the ground state, a concept further developed by Jencks, Leinhard, Wolfenden, and others [11, 12, 13, 14], often referred to as ‘transition state complementarity’. Experimentally, structure–function studies have been extremely valuable, as mentioned above. Most generally, the structure of an enzyme allows us to identify residues in its active site and propose the role of each residue based on its chemical properties. Typically, site‐directed mutagenesis experiments are then performed to confirm that these residues are important, based on the observation of large deleterious effects on catalysis from their mutation.

However, a comprehensive and quantitative understanding of catalysis could not be obtained from this structure–function paradigm, for several reasons. First, enzymes position reactants and catalytic groups to increase the probability of reaction, but a single, static structure cannot reveal the extent to which groups are positioned. Second, site‐directed mutagenesis compares wild‐type to mutant enzymes, rather than wild‐type enzymes to the corresponding solution reactions, which is the comparison that defines the overall rate enhancement; thus, site‐directed mutagenesis tests whether ablating a residue is deleterious but does not tell us how this residue provides catalysis when present. Third, and most fundamentally, free energy itself is a property arising from an ensemble of states, so the energetics that define reaction probabilities and catalysis cannot be determined from single, static structures.

In the following sections, we describe how we obtained atomic‐level information about enzyme conformational ensembles and used this information to dissect serine protease catalysis. In doing so, we discovered catalytic features that were not identified from decades of structure–function studies and achieved a previously unattainable quantitative accounting of catalysis by these enzymes.

## How to obtain atomic‐level ensemble information

How do we get the ensembles we need? The lab had been introduced to experimental approaches to obtain atomic‐level models for conformational ensembles by James (Jaime) Fraser, whom D.H. met at a conference in the Colorado mountains in 2013 and got to know on long hikes there. Jaime was one of Tom Alber's last graduate students before his untimely death, and together, Tom and Jaime developed (or redeveloped) noncryogenic X‐ray crystallography, also referred to as room temperature or multi‐temperature X‐ray crystallography (Tom was a student of Greg Petsko, a crystallographer and informal mentor of D.H., and both had early interests in protein dynamics and their possible functional roles. When D.H. was nearly finished with a perspective he wrote as a graduate student, he found similar ideas in a much earlier paper from Tom and Greg that was well ahead of its time [15, 16, 17, 18, 19]). Cryo‐freezing crystals became the norm in the 1990s after it was realized that freezing can reduce radiation damage, and > 90% of the Protein Data Bank X‐ray structures were obtained at cryogenic temperatures [20]. Fraser, Alber, and others also developed the program *Ringer* [21], which demonstrated statistical support for extracting ensemble information in X‐ray electron density maps, an idea Fraser extended into an automated software qfit [22, 23, 24, 25, 26] that creates ensemble models, in collaboration with Henry van den Bedem.

Filip Yabukarski, a postdoc in the Herschlag laboratory, worked collaboratively with Jaime on a model enzyme ketosteroid isomerase (KSI), using ensemble models obtained from noncryogenic X‐ray to show that KSI's active site residues were highly positioned, but not more so than other buried nonactive‐site residues [27]. These observations support the importance of positioning for catalysis but provide no indication of extraordinary positioning that is unique to the active site in this enzyme. Filip also found that KSI's general base, an aspartate (Asp), is more flexible than the other active site residues, apparently allowing this Asp general base to effectively transfer protons to different substrate positions that are separated by ~ 3 Å [27]. This optimized flexibility is achieved by an anion‐aromatic interaction, a less restrictive interaction than hydrogen bonds. Indeed, introducing a hydrogen bond to further restrict the positioning of Asp or a mutation to remove the constraints on the Asp both decrease catalysis, reminiscent of Goldilocks finding just the right porridge, chair, and bed.

Working with Tzanko Doukov at the Stanford Linear Accelerator (SLAC), Filip also developed approaches to advance our ability to rapidly obtain noncryogenic X‐ray structures and to do so at high resolution (< 1.5 Å) over a wide temperature range, up to 363 K, without incurring significant radiation damage [28].

**Fig. 3:**
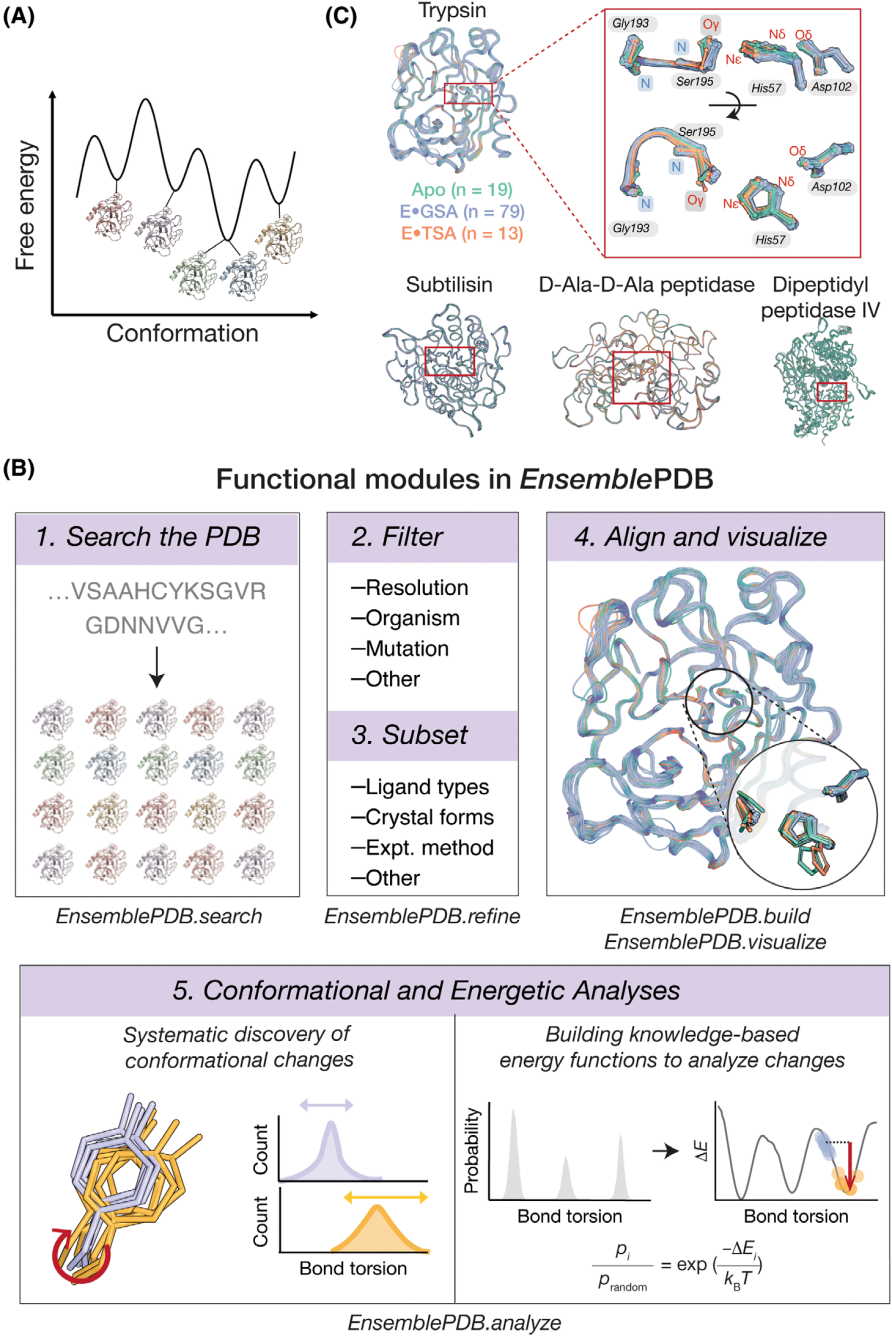
Building and analyzing pseudo‐ensembles from PDB structural data [61]. (A) Proteins crystallized under different experimental conditions can sample a range of low‐energy states on the conformational landscape [27]. (B) Functional modules in the *Ensemble*PDB package [4]. For detailed descriptions of the conformational and energetic analyses, see also Fig. 4 and associated text. See Movies S1–, S3 for pseudo‐ensemble examples. (C) Representative serine protease pseudo‐ensembles from each structural clan. Top: pseudo‐ensemble of trypsin, a serine protease in clan PA, with the number of structures in the apo, GSA‐bound, and TSA‐bound subensembles annotated. Bottom: pseudo‐ensembles of serine proteases across three additional structural clans—subtilisin (clan SB), dipeptidyl peptidase (clan SC), and D‐Ala‐*d*‐Ala peptidase (clan SE). Adapted from ref. [4] with permission.

Important for the investigations on serine proteases that followed, Filip showed that the ensemble of states obtained at room temperature was highly similar to those obtained by combining many snapshots of KSI cryogenic X‐ray structures into ‘pseudo‐ensembles’ [27]. Each frozen crystal has small variations—presumably arising from differences in crystallization conditions and captured during freezing—that appear to represent a range of low‐energy states on the protein's conformational landscape (Fig. 3A). This finding and prior results [e.g., [29, 30, 31]] supported the value of pseudo‐ensembles and emboldened us to build and analyze pseudo‐ensembles.

Since there were many repetitive structures for the same protein among the > 150 000 structures in the PDB, we set out to create a general tool to build and analyze pseudo‐ensembles. Figure 3B outlines the *Ensemble*PDB package, and Movies S1–, S3 depict some of these pseudo‐ensembles. Nevertheless, our goal was to go beyond descriptions and movies, to reveal *how and why* these motions occur—their energetic origins. Most structural comparisons use aggregated metrics based on distances between atomic coordinates, such as root‐mean‐square deviation (RMSD), and motions are typically described as domain rotations, loop closures, *etc*. Instead of these global descriptions, we systematically compared individual bond torsion angles across pseudo‐ensembles representing different reaction states, as bond rotation is the basic unit of noncovalent conformational changes. We could then quantify the energetic consequences of these rotations and the accompanying changes in additional interactions (Fig. 3B.5). *Ensemble*PDB provides functionalities that will allow others to carry out analogous analyses for their systems of interest, when there is sufficient structural coverage to build pseudo‐ensembles.

We thought the textbook enzyme serine proteases would be ideal for a deep mechanistic dive, and indeed, we found many serine protease structures in the PDB, including forms with ground state analogs (GSA) and transition state analogs (TSA) bound (Fig. 3C). GSAs are predominantly peptide inhibitors that favor the intact amide when bound, and TSA ligands form covalent tetrahedral adducts to the serine and have an oxyanion that sits in the enzyme's oxyanion hole. We curated and analyzed 1231 structures from 17 different serine proteases, which allowed us to carry out rigorous statistical tests and search for mechanistic generalities.

## An ensemble–function approach to decipher enzyme catalysis

We can break ensemble–function analysis of enzyme catalysis into four critical steps, as follows:

1. *Determining the rate enhancement provided by the enzyme*. Catalysis is, most fundamentally, a comparison of the enzymatic to the solution reaction rates. To begin, we need experimental measurements for those rates. There is a wealth of data from decades of physical organic and bioorganic chemistry, and Wolfenden famously obtained and curated nonenzymatic rate data for many biological reactions [see ref. [32] for a collated list]. Prior studies have also estimated the amount of catalysis from general base catalysis in solution [4, 33, 34, 35, 36]. These literature values from model reactions provide a reasonable estimate for the amount of catalysis that remains to be accounted for in serine proteases.
2. *Determining the enzymatic reaction path*. We infer changes along the enzymatic reaction path by building and comparing pseudo‐ensembles representing each reaction state. A sufficient number of structures in each pseudo‐ensemble is required to identify changes that are significant, and the number of structures needed will depend on the extent of change between states, the breadth of the ensemble states, and the quality of the structural models. In principle, with more structures, one can decipher even smaller conformational differences and calculate their energetic contributions; in practice, however, a large number of structures in an ensemble can also make tiny changes statistically significant, but those changes may include background noise, systematic errors, or real changes that may be too small to obtain reliable energetic information in Step 4 (below). For serine proteases, the absence of large‐scale conformational changes during the reaction minimized these complications. In addition, the availability of structures from multiple crystal forms of the same enzyme, multiple different serine proteases, and even many nonprotease enzymes with the same catalytic features provided strong support for the conclusions derived from the analyses of trypsin, the serine protease with the largest number of structures.
3. *Comparing the enzymatic reaction path to the uncatalyzed solution reaction*. To understand why the enzymatic reaction path provides a faster rate than in solution, we need analogous ensemble information about the solution reaction. Solution reactions that involve simple small molecules may be modeled by a combination of empirical and computational approaches. We first model the solution GS—for serine proteases, a peptide or amide in water—using molecular dynamics simulations and pseudo‐ensembles created from small‐molecule X‐ray structures. We then model the progression from the GS to the TS or intermediate state using quantum mechanical calculations. Empirical chemical knowledge, such as favored bond geometries, can also be used as a check on whether the calculations provide reasonable estimates. Comparing the enzymatic and nonenzymatic reaction paths reveals whether the enzyme follows a different path. The differences between the enzymatic and solution reaction states provide the foundation for determining the energetics of catalysis, as evaluated in Step 4.
4. *Creating knowledge‐based energy functions to estimate catalytic contributions*. Determining the amount of catalysis arising from the enzyme features requires energy functions that relate molecular configurations to their underlying energies. We create energy functions using a knowledge‐based approach, by collecting the geometric parameters of the interactions of interest across high‐quality protein and small‐molecule X‐ray structures and converting the probabilities into energies via the Boltzmann relationship (Fig. 3B.5). Support for the use of knowledge‐based energy functions comes from their agreement with NMR measurements and quantum mechanical calculations [e.g., [37, 38, 39, 40, 41]]. The robustness of the knowledge‐based energy functions used to evaluate serine protease features was tested by bootstrap analyses [4]; nonetheless, further testing the accuracy of knowledge‐based energy functions remains an important challenge for future studies.

Below, we summarize the analyses we carried out for serine proteases and the major conclusions.

## Applying ensemble–function to dissect the origins of serine protease catalysis

### Catalysis from positioning

Most catalytic proposals invoke precise positioning of the enzyme groups relative to the substrate [see table S1 of ref [4]]. Classic theoretical analysis by Page and Jencks [42] demonstrated that such positioning could provide enormous amounts of catalysis by reducing the entropic barrier to reaching the highly restricted TS; yet, it remained unclear how much catalysis was actually achieved by reduced conformational entropy. It might seem simple to determine this catalytic contribution since the activation entropy of a reaction can be obtained from the temperature dependence of a reaction rate. However, the change in activation entropy is fundamentally different from the change in the *conformational entropy* of the reactants, because the rearrangements of solvent and nonactive site groups represent the vast majority of the degrees of freedom in the system, leading to highly intertwined entropic and enthalpic changes (akin to entropy‐enthalpy compensation) that are difficult to deconvolute (In the classic account of the *Circe effect*, Jencks described this phenomenon as follows: “…This entropic advantage will appear in the observed free energy of activation even if the difference in entropy is masked by compensating entropy and enthalpy changes caused by solvation effects…Because there is no way to estimate the magnitude of these compensating changes *a priori*, the observed enthalpy and entropy of a particular reaction do not provide a direct measure of either the change in free energy resulting from the solvent effect or the intrinsic change in the entropy of the reactants.” [11]). In contrast, ensembles provide a direct manifestation of conformational entropy.

**Fig. 4:**
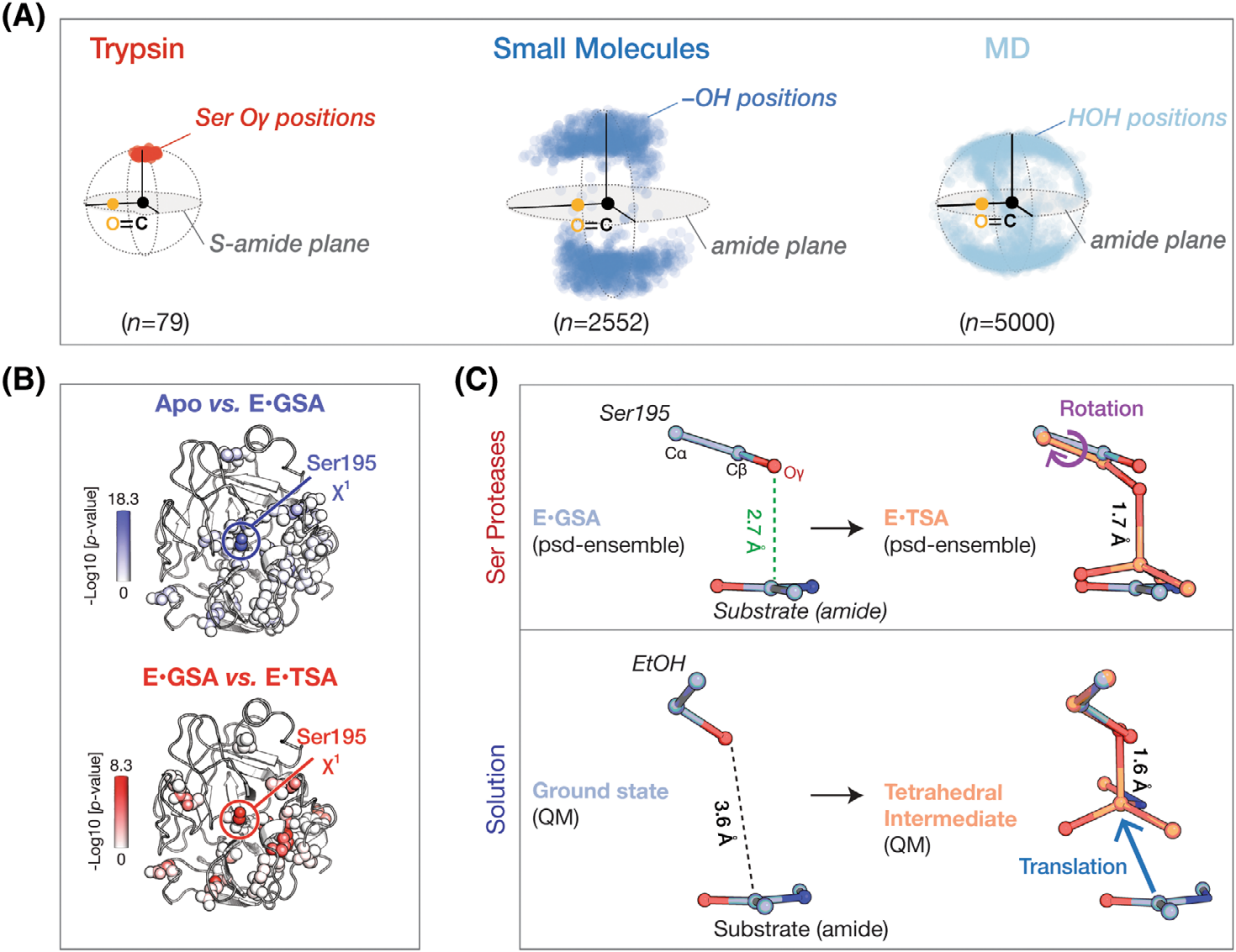
Trypsin pseudo‐ensemble reveals a highly positioned serine in the GS and a simplified and shortened reaction path compared to the uncatalyzed solution reaction. Adapted from ref. [4] with permission. (A) Spherical distributions of the nucleophile positions relative to the substrate amide group observed in the pseudo‐ensembles, with the electrophilic carbon placed at the origin and the substrate amide plane defining the *xy* plane. The colored circles represent positions that the oxygen nucleophile occupies (red: trypsin GSA‐bound pseudo‐ensemble; dark blue: small‐molecule hydroxyl oxygens that are in proximity to amide groups; light blue: water oxygens closest to the substrate amide in each MD snapshot). For visualization, only oxygen atoms on the back half of a sphere surrounding the reactive amide are shown. Gibbs entropy calculations for these distributions (not shown) gave a difference between the trypsin and small‐molecule distributions of ~ 2.0 kcal·mol^−1^, and the same value was obtained for the trypsin compared to the MD distribution. (B) Bond torsions that change significantly between the apo and GSA‐bound state (blue) and the GSA to the TSA‐bound state (red), mapped onto the trypsin structure. A total of 428 torsion angles were tested (all buried residues with relative solvent accessibility of < 0.25); the bonds that change significantly are shown as spheres and colored by their significance [−log_10_(*P*‐value)]. (C) The changes along the reaction path on serine proteases (inferred from pseudo‐ensembles) *versus* in solution (calculated by QM). The center structure from each trypsin pseudo‐ensemble is shown [E•GSA: 3M7Q [62]; E•TSA: 1TPP [63]] for clarity. The purple arrows indicate the rotation of the catalytic serine side chain in the trypsin reaction, and the blue arrow represents the dominant translation motion in the solution reaction.

In GSA‐bound pseudo‐ensembles of serine proteases, the nucleophilic serine oxygen atom is remarkably constrained with respect to the substrate amide group compared to the reactants in the GS of the solution reaction (Fig. 4A). QM calculations indicated that the reactants are constrained in the optimal orientation for bond formation on the enzyme. While suggestive of a catalytic contribution, this result was qualitative. What we needed to do next was to quantify the conformational entropy of the reacting groups with respect to one another on the enzyme and compare it to that in solution.

Gibbs formulated entropy as the extent of spread of a distribution [Gibb's entropy [43]; *S* = $-k_{B}\sum p_{i}\ln p_{i}$, where $k_{B}$ is the Boltzmann constant and $p_{i}$ is the probability of state *i*]. Thus, we can calculate conformational entropy of the reactants by forming a grid of cubes in 3D space and counting the number of observations in each cube. We performed multiple controls varying cube size and subsampling the data to ensure the robustness of the resulting entropy estimates and to obtain error estimates. We found that the conformational entropy of reactants on the enzyme is ~ 2.2 kcal·mol^−1^ lower than that in the solution ground state, corresponding to a ~ 40‐fold higher probability of reaction. Thus, enzyme positioning can provide a substantial catalytic advantage, even compared to hydrolysis in bulk water at 55 m. This positioning corresponds to an effective molarity (EM) of 2200 m relative to the standard state of 1 m water. It will be of great interest to obtain EM values for a variety of enzymatic reactions to assess the generality of positioning as a catalytic feature, to dissect the structural and binding interactions that constrain the reacting groups to achieve a high EM, and also to compare these values to those of designed enzymes.

### What happens on the enzyme's reaction path

While positioning in the ground state provides catalysis, motions are needed for the reactants to progress along the reaction path. We therefore compared pseudo‐ensemble distributions of each bond torsion in the apo, GSA‐bound, and TSA‐bound states (Fig. 4B). This unbiased comparison revealed a highly significant, ~ 15° rotation of the catalytic serine sidechain (*χ*_1_) from the GSA to the TSA‐bound state that brings the reactants ~ 0.3 Å closer to each other, thereby facilitating bond formation (Fig. 4C). We found the same rotation within and across multiple serine protease structural families, suggesting that nature has evolved this reaction path repeatedly [4].

Comparisons of the enzymatic and solution reaction paths revealed fundamental differences. Unlike the enzymatic reaction, the solution reaction does not involve a bond rotation. Rather, the majority of changes along the solution path are achieved via relative translational movement of the reacting water and amide, matching the simplest expectation for a bimolecular reaction. These comparisons also revealed that the reactant distance in serine proteases (~ 2.7 Å) is substantially shorter than in the solution ground state (3.6 Å, the ideal van der Waals interaction distance; Fig. 4C).

These ensemble–function analyses suggest that serine proteases remodel the reaction path to make it shorter and simpler, replacing much of the three‐dimensional translation with a one‐dimensional bond rotation that is aligned to the reaction path. By doing so, the enzyme increases the probability of reaction by limiting the unreactive states while maintaining freedom to progress along the reaction path.

### Quantifying catalytic contributions from serine protease features: the catalytic ledger

**Fig. 5:**
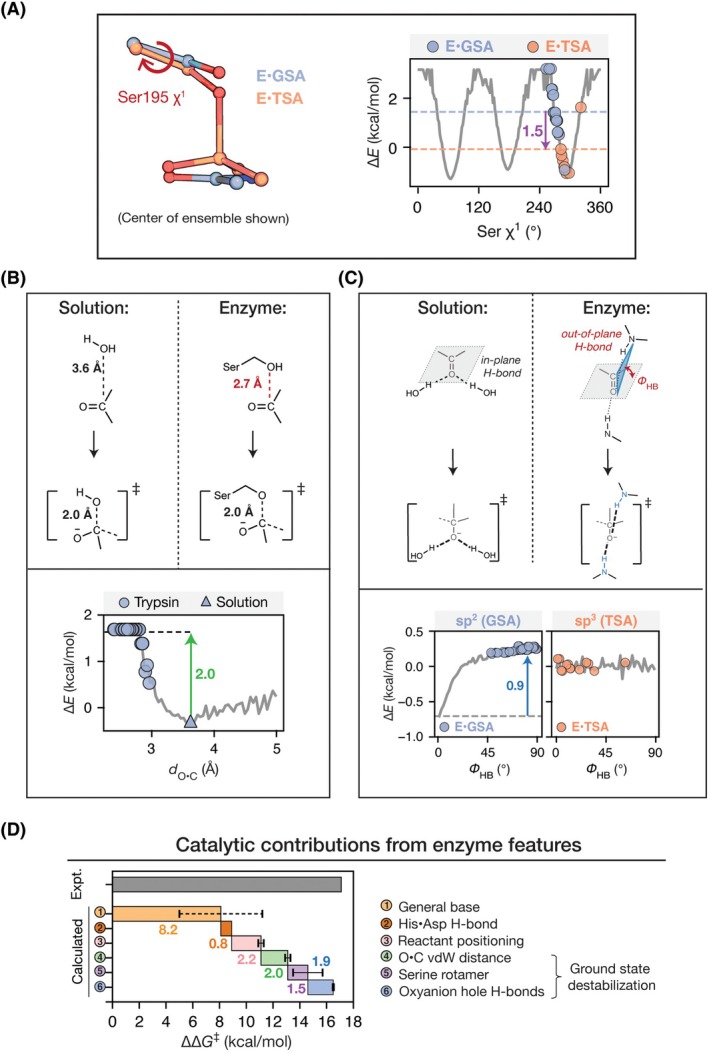
Ensemble–function energy analyses to quantify catalytic features from serine proteases. Adapted from ref. [4] with permission. (A) Catalytic contribution from the rotation of the catalytic serine from the GSA to the TSA‐bound states, quantified by a knowledge‐based energy function of serine *χ* ^1^ built from 41 237 serine residues across high‐resolution PDB structures. Catalytic serine *χ* ^1^ values from the trypsin pseudo‐ensembles were mapped on the energy function (GSA‐bound: blue; TSA‐bound: orange; dashed lines indicate their mean values). (B) Catalytic contribution from a shorter O•C distance between the reacting atoms on the enzyme compared to in solution, quantified by a knowledge‐based energy function of O•C van der Waals interaction built from 2552 amide•hydroxyl interactions collected from the Cambridge Structural Database [64]. (C) Catalytic contribution from suboptimal GS hydrogen bonds in the oxyanion hole that are relieved in the transition state. Solution amide•carbonyl hydrogen bonds prefer an in‐plane orientation, whereas the enzymatic ground state has out‐of‐plane (destabilized) oxyanion hole hydrogen bonds, as illustrated by the large dihedral angle, Φ_HB_, between the gray and the blue planes. The energetics were quantified by knowledge‐based energy functions of Φ_HB_, built from 60018 small‐molecule amide•carbonyl hydrogen bonds (with sp^2^ acceptors, mimicking the GS) and from 5823 hydrogen bonds between amide and sp^3^ oxygens (mimicking the TS). (D) The sum of the energetic contributions from the identified catalytic feature accounts for trypsin's total rate enhancement from trypsin within error [17.1 kcal·mol^−1^ from experiment (gray bar) *vs*. 16.6 kcal·mol^−1^ calculated (colored bars, with colors corresponding to each catalytic feature)]. The error bar for general base catalysis (dashed line) indicates lower and upper ranges; all other error bars (solid lines) indicate standard deviations from pseudo‐ensemble data points, as described in [4].

While significant differences were uncovered between the enzymatic and solution reaction paths, simply seeing the changes does not tell us whether they contribute to a faster rate and, if so, by how much. To determine the energetics associated with these differences, we derived knowledge‐based energy functions for each feature and mapped pseudo‐ensemble observations onto the corresponding energy function (Fig. 5). Using this empirical approach, we found that a substantial amount of catalysis is achieved by the newly discovered enzyme features—a bond rotation (Fig. 5A), a shorter‐than‐ideal reactant distance (Fig. 5B), and a pair of suboptimal hydrogen bonds at the oxyanion hole that were previously described and were supported by our analyses [Fig. 5C; [44, 45]]. Remarkably, each of these catalytic features is explained and quantified by basic physical and chemical properties of molecular interactions taught in introductory‐level undergraduate courses, underscoring the value of using the natural language of physics and chemistry to dissect the behavior of biomolecules.

Knowledge‐based energy functions revealed that the catalytic serine occupies a semi‐eclipsed bond torsion in the GS that is ~ 1.5 kcal/mol destabilized from the local energy minimum, and the ~ 15° rotation along the reaction path relieves this destabilization in the TS, thereby lowering the reaction barrier (Fig. 5A). The short distances on the enzyme represent a sterically hindered state that is ~ 2.0 kcal·mol^−1^ destabilized compared to the solution distances; again, this destabilization is relieved in the TS (Fig. 5B). The oxyanion hole hydrogen bonds are orthogonal to the carbonyl in the GSA, rather than occupying a favored in‐plane geometry, and while progressing to the TS, charge accumulation on the oxyanion removes this orientational preference to relieve the destabilization, contributing an additional 1.9 kcal·mol^−1^ to catalysis from the pair of hydrogen bonds (Fig. 5C).

Together, the chemical and conformational features at the serine protease active site fully account for catalysis by serine proteases (Fig. 5D). Other than the histidine general base and the His‐Asp hydrogen bond, serine proteases do not enhance reactivity relative to water through the intrinsic chemistry of active site groups. Rather, these enzymes create a GS complex with locally destabilized interactions that are relieved in the TS, an energetic gradient that facilitates motions along the reaction path.

## Generalities, conclusions, and future directions

**Fig. 6:**
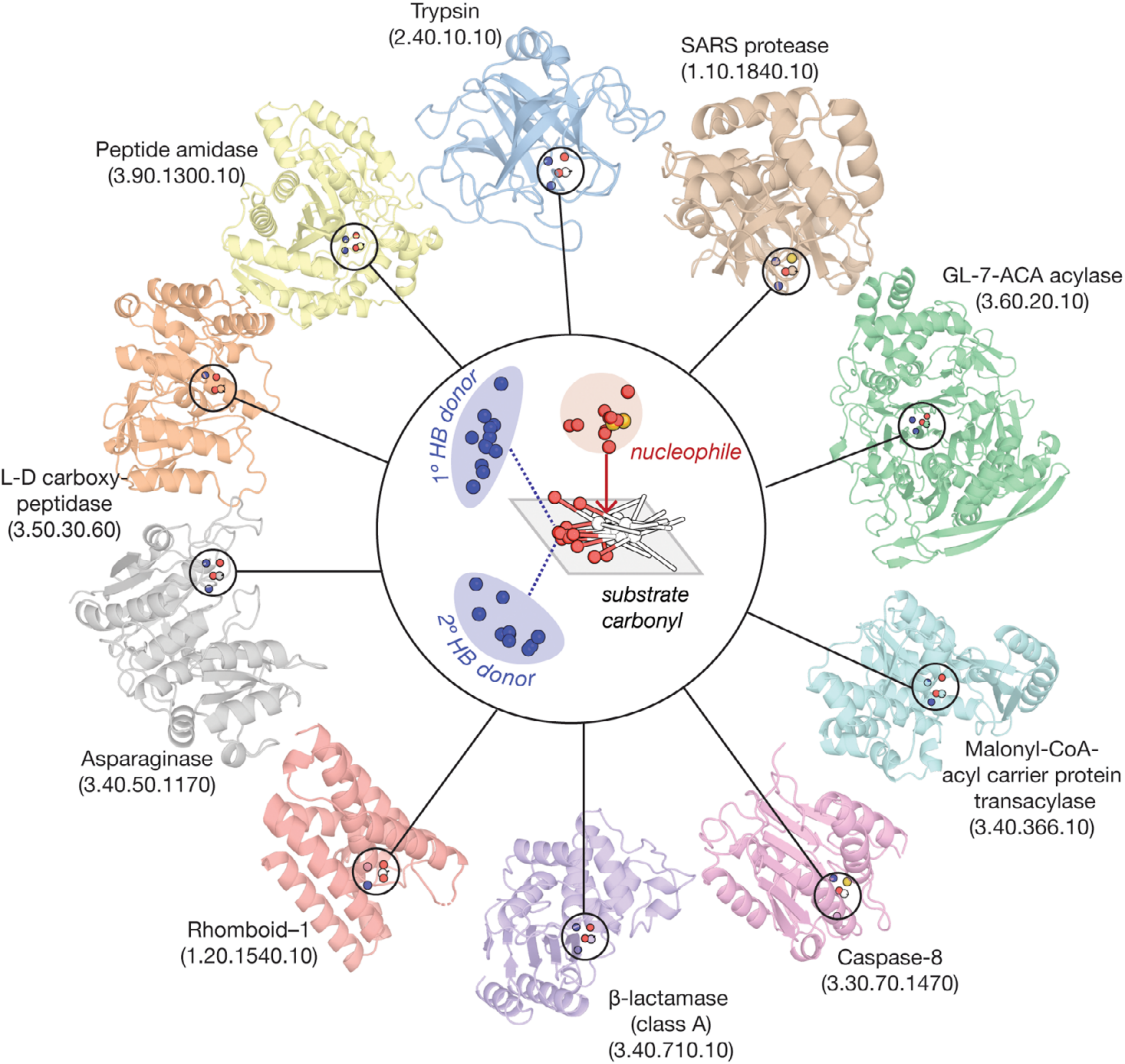
A common catalytic conformation in enzymes across distinct structural folds. *Inner circle*: The active sites of representative enzymes across 10 different structural folds in their GSA‐bound states, locally aligned to a reference trypsin structure [PDB: 3M7Q [62]]. *Outer circle*: The global structures of the enzymes from the 10 different structural folds. Enzyme names are shown beside them, along with the identifiers for their structural classification (in parentheses) as defined in the CATH database [65]. From ref. [4] with permission.

The catalytic triad and oxyanion hole of serine proteases are classic examples of convergent evolution, as analogous groups were found in proteases across multiple structural families [3]. We found that serine proteases also converge to use the same catalytic strategies of positioning and ground state destabilization [4]. Further expanding our search for these features in additional enzymes catalyzing nucleophilic addition reactions, we found them in a total of 30 different proteases (*n* = 17) and nonprotease enzymes (*n* = 12), spread across 12 distinct structural folds (Fig. 6). Thus, nature seems to have discovered catalytic features that can be inserted into multiple protein templates and used in a broad swath of reactions, perhaps reflecting their evolvability, that is, a high probability to acquire these catalytic features on an evolutionary timescale.

Despite decades of effort, successes in enzyme engineering have largely relied on exploiting the catalytic promiscuity of existing enzymes [46, 47, 48] and on directed evolution from natural or designed scaffolds [49]. Even after rounds of optimization, the most recent designed enzymes still fall short of the rate enhancements of their natural counterparts [e.g., [50, 51]]. Newer algorithms have substantially improved in generating viable sequences that fold into protein scaffolds around a designated active site [52, 53]. The features we discovered here and additional enzyme features yet to be discovered by ensemble–function investigations could provide a library of catalytic features and motifs grounded in physical mechanisms that will guide the design of more effective enzymes.

The approaches established in this work unlock exciting opportunities to derive deep and quantitative mechanistic models for enzyme catalysis and additional aspects of protein function. Using pseudo‐ensembles, noncryogenic X‐ray crystallography, and potentially cryo‐electron microscopy at sufficiently high resolution, ensemble–function analyses can be performed for many additional important enzymatic reactions and for designed enzymes. An exciting possibility is to extend this framework to dissect protein functions that involve more extensive conformational changes, including allostery and the action of molecular motors, which will allow researchers to move beyond descriptions of conformational changes to the fundamental energetic underpinnings responsible for these changes.

The ensemble–function approach provides mechanistic models that are grounded in the most basic of chemical concepts—bond torsion angles, van der Waals interactions, hydrogen bonds, etc.*—*thus allowing students and researchers to connect complex biological functions to these basic concepts. We envision a revised and integrated undergraduate chemistry and biology curriculum that introduces these core concepts in early chemistry courses and returns to them in higher‐level biology and biochemistry courses. This approach would simultaneously enrich understanding of core chemical principles and deepen understanding of biological processes, enhancing mastery by connecting these disciplines.

The cornerstone of the ensemble–function framework is the language of chemistry and physics that links molecular configurations to energetics, and this linkage has broad implications for the development of predictive algorithms for biomolecules. The ability to quantitatively predict functions—protein•ligand and protein•protein binding affinities, catalytic rates, etc.—would provide unprecedented biological insight and generate remarkable biomedical applications. Biomolecular function emerges from molecular interactions, yet the energetics underlying these interactions have not been emphasized in the development and evaluation of current deep learning models. Biomolecular modeling has instead relied on RMSDs and analogous aggregate metrics for model training and evaluation, so that models are encouraged to match atoms in overall space, but not to match the underlying physical interactions and their probabilities. Indeed, a systematic evaluation of the molecular interactions predicted by state‐of‐the‐art structure prediction models revealed widespread conformational biases, with over 30% incorrect noncovalent interaction partners and networks [54]. Developing new training and evaluation approaches grounded in physics and chemistry will enable AI models to learn the energetic rules that govern biomolecules, allowing them (and us) to achieve the greatest predictive power.

Biochemistry, biophysics, and structural biology have experienced a long and fruitful structure–function era. As with all good things, a time comes to move on. We are excited by the forthcoming ensemble–function era and the breakthroughs that will follow.

## Conflict of interest

The authors declare no conflict of interest.

## Author contributions

DH and SD wrote the manuscript and SD prepared figures.

## Supporting information


**Movie S1.** Pseudo‐ensemble of carbonic anhydrase 2, built by aligning 652 cryogenic X‐ray structures on Cα atoms.

**Movie S2.** Pseudo‐ensemble of myoglobin, built from 94 cryogenic X‐ray structures on Cα atoms.

**Movie S3.** Pseudo‐ensemble of lysozyme, built by aligning 55 cryogenic X‐ray structures on Cα atoms.
